# Transposable element-derived sequences in vertebrate development

**DOI:** 10.1186/s13100-020-00229-5

**Published:** 2021-01-06

**Authors:** Ema Etchegaray, Magali Naville, Jean-Nicolas Volff, Zofia Haftek-Terreau

**Affiliations:** Institut de Genomique Fonctionnelle de Lyon, Univ Lyon, CNRS UMR 5242, Ecole Normale Superieure de Lyon, Universite Claude Bernard Lyon 1, 46 allee d’Italie, F-69364 Lyon, France

**Keywords:** Transposable elements, Vertebrates, Development, Genetic innovation, Exaptation, Genome evolution

## Abstract

Transposable elements (TEs) are major components of all vertebrate genomes that can cause deleterious insertions and genomic instability. However, depending on the specific genomic context of their insertion site, TE sequences can sometimes get positively selected, leading to what are called “exaptation” events. TE sequence exaptation constitutes an important source of novelties for gene, genome and organism evolution, giving rise to new regulatory sequences, protein-coding exons/genes and non-coding RNAs, which can play various roles beneficial to the host. In this review, we focus on the development of vertebrates, which present many derived traits such as bones, adaptive immunity and a complex brain. We illustrate how TE-derived sequences have given rise to developmental innovations in vertebrates and how they thereby contributed to the evolutionary success of this lineage.

## Background

Transposable elements (TEs) were discovered by Barbara McClintock in the 1940s and described as moving DNA sequences that can cause genomic instability [1]. As she was able to link TE activity with variations in maize kernel colors, she coined them “controlling elements”, underlying their apparent involvement in gene regulation. TEs are nowadays known to be major components of genomes and have been found in every species that has been looked at, including prokaryotes, protists, fungi, plants and animals [2–4].

TEs are classified into two main classes according to their transposition mechanism [5, 6]. The transposition of retrotransposons (class I TEs) occurs through the reverse transcription of an RNA intermediate into a cDNA molecule that is subsequently inserted into a new locus [7, 8]. This replicative transposition process, a “copy-and-paste” mechanism called retrotransposition, leads to the expansion of the retroelement family in the host genome. Retrotransposons gather both Long Terminal Repeat retrotransposons (LTRs), with flanking repeated sequences in direct orientation necessary for the expression and integration of the element, and non-LTR retrotransposons, also called Long Interspersed Nuclear Elements (LINEs). Autonomous retrotransposons encode a reverse transcriptase (RT) and other proteins necessary for integration (an integrase for LTRs and an endonuclease for LINEs) and other aspects of transposition [7–9]. In contrast, non-autonomous retrotransposons, including Short Interspersed Nuclear Elements (SINEs) that are mobilized by autonomous non-LTR retrotransposons, do not encode any proteins and rely on those produced *in trans* by autonomous elements to transpose [10, 11]. DNA transposons (class II TEs) do not require the reverse transcription of an RNA intermediate for their transposition [12]. They mostly use a “cut-and-paste” mechanism, the TE copy being excised from its original locus and integrated elsewhere into the genome. Many DNA transposons, including the widespread DDE transposon family, classically encode a transposase (with the DDE motif forming its active site in DDE transposons) and are flanked by Terminal Inverted Repeat (TIR) sequences that are bound by the transposase for excision and integration [9, 12]. Other types of DNA transposons include Helitrons [13, 14], which are rolling-circle DNA transposons with no TIRs encoding a helicase, and Polintons/Mavericks [15, 16], which are self-synthesizing DNA transposons with long TIRs encoding a DNA polymerase. Non-autonomous elements called Miniature Inverted Repeat Transposable Elements (MITEs) are mobilized *in trans* by related autonomous DNA transposons [12].

Each species genome is characterized by a specific composition in TEs, both quantitatively and qualitatively. For instance, the genome of the maize *Zea mays* is composed of nearly 85% of transposable elements [17], whereas the genome of the yeast *Saccharomyces cerevisiae* contains less than 4% of TEs [18]. In unicellular organisms, the genome of *Trichomonas vaginalis* contains almost exclusively DNA transposons, while almost only retrotransposons are found in *Entamoeba histolytica* [19, 20]. A marked variability in TE content and diversity has been also observed among vertebrates [21]. Indeed, the genomic amount of TEs ranges from 6% in the pufferfish *Tetraodon nigroviridis* up to 55% in the zebrafish *Danio rerio*. Some groups of TEs are found in most vertebrate species (LINE retrotransposons or Tc-Mariner DNA transposons for instance), whereas others are restricted to certain vertebrate sublineages and absent from others, such as the DIRS and Copia retrotransposons that are present in fish and amphibians but absent from mammals and birds [21].

**Fig. 1:**
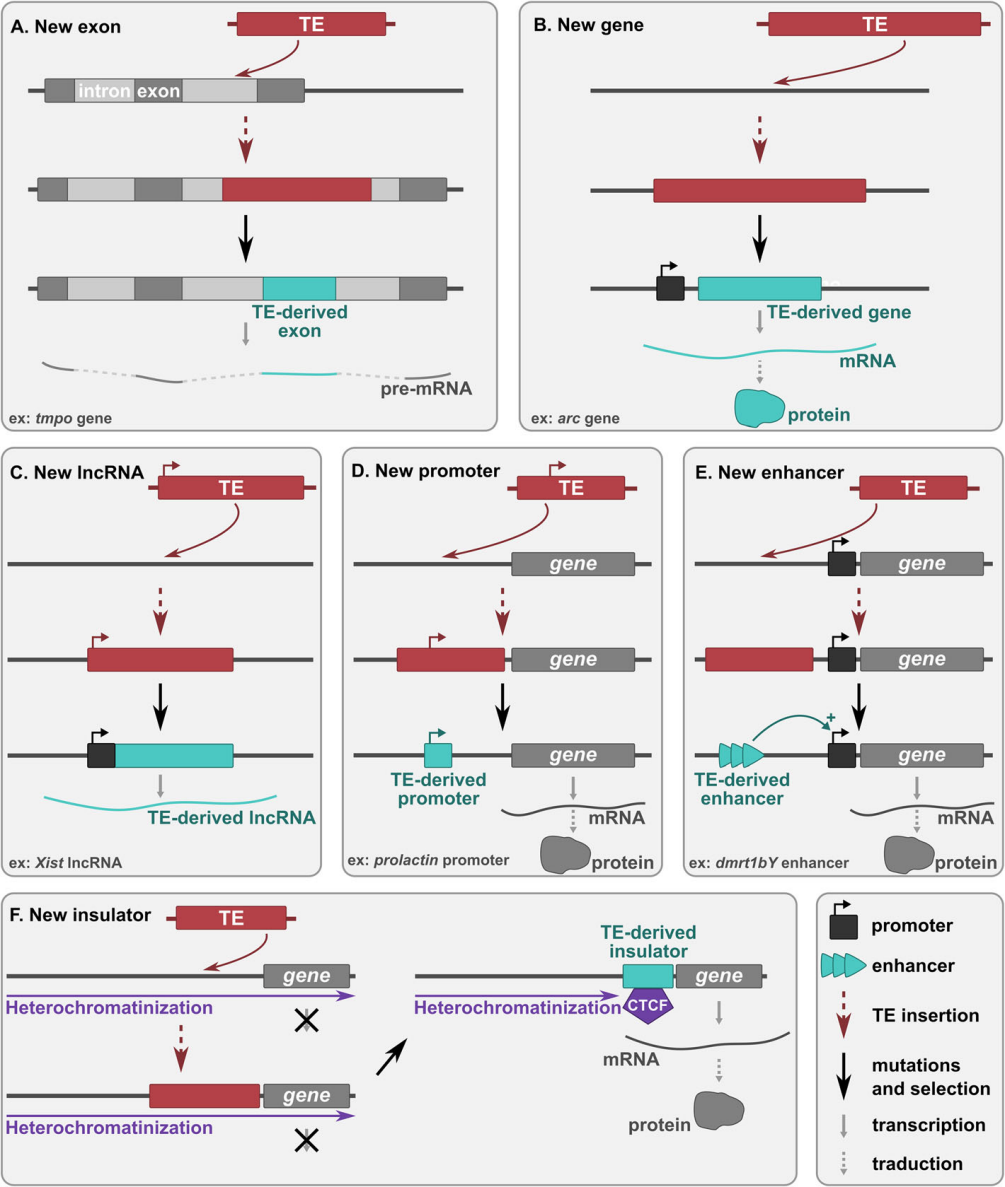
Adaptive mechanisms of TE-derived sequences evolution leading to developmental innovations. After the insertion of a TE: **a** in an intron of a protein-coding gene, part of the TE can give rise to a new exon (exonization). Splicing sites can either be directly present in the TE sequence or can be acquired by mutations. **b** part of the TE can form a new host gene and be transcribed from either a flanking host promoter or a promoter derived from the TE sequence itself. **c** the TE can form a new long non-coding RNA (lncRNA) gene and be transcribed from either a flanking host promoter or a promoter derived from the TE sequence itself. **d-e** in the upstream region of a coding or RNA gene, the TE can form a new promoter (D) or enhancer (this model also works for TE-derived silencers) (**e**). **f** the TE can form an insulator region, which recruits the CCCTC-binding factor (CTCF) and blocks heterochromatin spreading, allowing the expression of downstream sequences. Red boxes correspond to TEs and blue boxes to exapted TE sequences

Most TE insertions are thought to be either neutral or deleterious, depending on the context of the genomic region where they are inserted. TE insertions can be deleterious for instance by disrupting open reading frames (ORFs) or by altering gene transcriptional regulations. However, and despite their “selfish” characteristics, TEs are subject to the drift-selection balance and can be positively selected if they are beneficial to the host [12]. Indeed, some insertions have been shown to play a positive role in species evolution by contributing to new regulatory and coding sequences (Fig. 1) [22–28]. Such a recruitment by the host to fulfil useful functions is called exaptation or molecular domestication. The ability of TE sequences to give rise to evolutionary innovations has been more and more documented in the past years and becomes of growing interest, helped by the recent technological developments in genome sequencing and gene expression profile analysis. The structural and functional characteristics of different TE families might confer them with different potential to be exapted. TEs can contain different functional ORFs encoding proteins with various properties such as endonucleases, integrases, transposases, reverse transcriptases and other proteins with DNA/RNA/protein-binding domains, and diverse transcriptional regulatory sequences such as promoters or enhancers. For example, LINE L1 elements contain an internal RNA polymerase II promotor and encode beside an RT an RNA-binding protein and an endonuclease; SINEs in contrast do not carry any ORF and have an RNA polymerase III promoter; LTR retrotransposons present transcriptional regulatory sequences in their long terminal repeats and generally encode an integrase, a protease, a RNase H and a structural protein called GAG in addition to their RT, with an additional Envelope gene that Endogenous Retroviruses (ERVs) have occasionally kept from their infectious ancestors; DNA transposons can among others code for transposases, helicases and DNA polymerases. These functional ORFs and regulatory sequences can be reused to the host benefits. The mobilome can thus be regarded as an evolutionary toolbox, as TEs bring with them in host genomes sequences encoding proteins able to bind, replicate, cut, rearrange or degrade nucleic acids, and to associate with and modify other proteins, among other biologically relevant properties.

Vertebrates constitute a geographically widely expanded taxonomic group that appeared more than 500 million years ago and has colonized almost all ecological environments [29]. The emergence of vertebrates represents a major evolutionary transition. This group has acquired many derived traits, namely: a unique nervous system composed of a complex brain with forebrain, midbrain and hindbrain specialized regions, and cranial nerves, spinal cord and ganglia; the sensory placodes and the sensory organs they give rise to (olfactory bulbs, vestibular apparatus and otic placode for example); the neural crest, which develops into cranium, branchial skeleton and sensory ganglia; a complex endocrine system allowing the apparition of new hormones and new organs such as the placenta; bones and cartilages contributing to the skull, jaws and vertebrae; paired appendages; adaptive immunity [30–32]. These novelties, which subsequently diversified in different sublineages, have contributed to the evolutionary success of vertebrates, allowing them to improve the sense of and the move in their environment, to develop new organs and complexify them, and to turn to extensive predation.

At the origin of vertebrates, two events of whole genome duplications allowed a massive expansion of the gene repertoire [33]. However, the sole emergence of paralogous genes may not explain all the innovations that appeared, and it has been also proposed that regulatory divergence might account for major organismal diversification [34, 35]. Accordingly, the analysis of the genome of the cephalochordate amphioxus, a sister outgroup species of vertebrates, has underlined the specialization of gene expression and the complexification of gene regulation during invertebrate to vertebrate transition, mainly due to the recruitment of new regulatory networks [36]. The precise understanding of the genetic and evolutionary mechanisms underlying this transition is of particular interest, and we propose to explore the role of TEs in this context. Several examples of TE recruitment events crucial for vertebrate development have been documented in the last years. In this review, we discuss the different mechanisms through which TE-derived sequences have played a role in vertebrate genome evolution. We focus on selected examples illustrating the innovative potential of transposable elements as a source of new protein-coding sequences, new small and long non-coding RNA genes and new regulatory elements having driven the evolution of vertebrate development.

## TE-derived sequences as new protein-coding sequences

### TE exonization

Inserted TE sequences can occasionally be recruited as new exons of pre-existing genes, a process called TE exonization (Fig. 1a). Exonization is defined as the formation of a novel exon from an intronic or intergenic sequence carrying splicing sites. Such new exons can be protein-coding but might also constitute new 5′ or 3′ untranslated regions with possible regulatory functions.

TE exonization is not an anecdotal process and has been largely documented in mammals and other vertebrates, where it occurs more frequently than in non-vertebrate species [37–39]. In the human genome, among 233,785 exons, more than 3000 (~ 1%) are derived from TEs [37, 40]. Among them, about 1640 correspond to Alu SINE elements, 640 to LINEs, 310 to MIRs (Mammalian-wide Interspersed Repeats, SINE elements), 300 to LTRs and 230 to DNA transposons [37]. Human exonized TEs are generally alternatively spliced, allowing protein variability [41–43]. It was also hypothesized that many TE-derived exons act as post-transcriptional gene regulators instead of being part of the protein-coding sequence itself [40]. The prevalence of Alu elements as TE-derived exons can be linked not only to their high copy number -with 1200,000 copies, they constitute as much as 10% of the human genome [44], but also to the fact that Alu sequences contain many potential splicing sites [45]. Alu elements indeed present up to ten 5′ and thirteen 3′ cryptic splicing sites that can be activated into functional splice sites through mutations or modifications such as adenosine-to-inosine RNA editing [38, 41]. Alu exons often modulate translational efficiency and can lead to lineage-specific regulations of gene translation [46]. Alu exonization can also cause genetic diseases in human such as the Alport syndrome, which is characterized by progressive renal failure, hearing loss and ocular abnormalities [47]. LINEs and to a lesser extent LTR retroelements can be exonized too [48, 49].

Exonization of intronic insertions is influenced by multiple factors. In the human genome, exonization is promoted by large intron size, high intronic GC content, and, importantly, by the presence of young transposable elements, in particular close to transcription starting sites [50]. These factors might contribute to a decrease of RNA polymerase II elongation rate and to a reduction of spliceosomal efficiency, allowing an increase of the “window of opportunity” for spliceosomal recognition and thus for exonization. Other mechanisms inhibit Alu exonization. It has been shown in human that the RNA-binding protein hnRNP C prevents Alu exonization by avoiding the binding of splicing factor U2AF65 to Alu cryptic exons, thus blocking Alu splicing sites; this prohibits Alu exon inclusion that would potentially lead to the formation of aberrant transcripts [51]. The binding of hnRNP C to Alu RNA is highly dependent on two poly(U) tracts present in Alu sequences inserted and transcribed in antisense orientation compared to the gene. These poly(U) arise from the antisense transcription by the gene promoter of the Alu terminal poly(A) and the internal poly(A) linker separating the two arms of Alu sequences (Alu are dimeric elements). Point mutations in these Alu poly(U) sequences are sufficient to impair the binding of hnRNP C [51]. Thus, the accumulation of mutations preventing hnRNP C binding can favor Alu exon inclusion.

Some examples illustrate well how intronic TEs can drive transcriptome and proteome diversification through the formation of lineage- and tissue-specific alternative exons. The vertebrate *lamina-associated polypeptide 2* gene (*tmpo* for *thymopoetin*) encodes several membrane protein isoforms including LAP2β suggested to control nuclear lamina dynamics at the nuclear periphery by binding specifically to B-type lamins. Another isoform, the mammalian-specific LAP2α protein, has a domain derived from the *gag* ORF of a DIRS1-like retrotransposon [52]. Unlike other isoforms, LAP2α is a non-membrane protein that binds to A-type lamins in the nucleoplasm [53]. This isoform is implicated in nuclear organization dynamics during the cell cycle [54, 55]. A mutation in the TE-derived domain of LAP2α has been associated with dilated cardiomyopathy in humans [56].

In mammals, the gene *prl3c1* belonging to the prolactin gene family encodes a cytokine expressed in uterine decidua and implicated in the establishment of pregnancy. In rodents, this gene has acquired a novel transcript variant in a common ancestor of the house mouse *Mus musculus*, *M. spretus* and *M. caroli* through the insertion of a composite TE into its first intron [57]. The inserted TE, which consists of an LTR element interrupted by a LINE, gave rise to an alternative promoter and an alternative first exon. In contrast to the “classical” transcript, the new variant is expressed in the Leydig cells of the testis. The variant protein shows a different intracellular localization and modulates the growth of testes and their capacity to produce testosterone and sperm. Such a TE co-option might contribute to the diversity of testicular development and functioning.

The *rtdpoz-T1* and *rtdpoz-T2* retrogenes, specifically expressed in testis and in the developing embryo in rat, and supposed to encode nuclear scaffold proteins functioning as transcription regulators, have multiple exons deriving from TE sequences [58, 59]. For example, *rtdpoz-T1* has 5 out of 8 exons and an alternative polyadenylation signal that are derived from various TEs, mainly L1 and ERVs. These TE-derived exons may be implicated in the translational regulation of these transcripts, notably through the formation of upstream ORFs [59].

The vertebrate insulin-like growth factor 1 (IGF-1) is a hormone involved in the development and growth of many tissues. IGF-1 plays a role for instance in synapse maturation and skeletal muscle development. Three isoforms of IGF-1 are known, IGF-1Ea, IGF-1Eb and IGF-1Ec [60]. The IGF-1Ea isoform is conserved among vertebrates, whereas the two others are mammal-specific and coincide with the insertion of a MIR-b SINE element that allows the formation of a fifth exon [61]. This fifth exon adds a disordered tail to IGF-1, which is highly suspected to be the source of post-translational modifications and regulatory functions. This allows a lineage-specific regulation of IGF-1.

Finally, the exonization of an Alu-J SINE element has been linked to the evolution of hemochorial placentation in anthropoid primates [62]. Hemochorial placentation is a placental implantation specific to rodents and higher order primates. In this type of placenta, the maternal blood is separated from the fetal blood by only one barrier, the chorion. This may optimize nutrient and gas exchange but makes the immune tolerance more challenging. The chorionic gonadotropin (CG) is a heterodimeric glycoprotein hormone formed by an alpha subunit, the glycoprotein hormone alpha (GPHA), and a beta subunit CGB [63]. CG is involved in the regulation of ovarian, testicular and placental functions. An Alu-J is inserted in the *gpha* gene in anthropoid primates, and its alternative exonization induces the formation of a GPHA isoform called Alu-GPHA that contains an additional N-terminus [62]. This isoform is only expressed in chorionic villus tissues and placenta, while the GPHA isoform without the Alu is expressed in other tissues. In human, the heterodimer Alu-hCG formed with the subunit Alu-GPHA shows a longer serum half-life and has a better trophoblast invasion activity compared to hCG, allowing the improvement of placenta implantation and invasion.

### TE molecular domestication to form new protein-coding genes

TEs can give rise to new functional host genes, a process known as molecular domestication (Fig. 1b). In the human genome, more than hundred protein-coding genes are thought to be derived from TEs [64, 65], representing about 0.5% of the complete set of human protein-coding genes. For example, the mammalian centromere protein B (CENP-B) is derived from the transposase of a pogo-like DNA transposon [66, 67]. Like its transposase ancestor, this protein is able to bind DNA. CENP-B is involved in centromere formation during both interphase and mitosis, and directs kinetochore assembly. Ty3/gypsy LTR retrotransposons have given rise to several multigenic gene families including the Paraneoplastic (PNMA, also called *Ma* genes, 15 genes), MART (12 genes) and SCAN families (56 genes) [68–71]. Overall, at least 103 genes derived from GAG proteins of Gypsy LTR retrotransposons have been identified in mammalian genomes, 85 being present in the human genome.

#### TE domestication and lymphocyte development

Two important TE-derived proteins in jawed vertebrates are RAG1 and RAG2 (Recombination Activating Gene 1 and 2) that together catalyze the V(D)J somatic recombination, a mechanism essential for the establishment of the vertebrate immune repertoire [72]. This genetic recombination, which takes place in developing lymphocytes, is at the basis of the adaptive immune system, since it allows the formation of diverse antibodies and T-cell receptors capable of specifically recognizing a great variety of pathogens. Pathogen recognition is ensured by the antigen-binding domain, which is encoded after assembling gene segments called variable (V), diversity (D) and joining (J). The joining of different V, D and J segments generates, in association with additional mutational processes, the great diversity of antibodies that can be produced by a jawed vertebrate.

RAG1 and RAG2 lymphoid-specific endonucleases are key enzymes for this somatic recombination. Both proteins associate as a recombinase to introduce double-strand breaks in DNA at recombination signal sequences (RSSs) that frame each V, D and J gene segment. This DNA cleavage resembles the transposition mechanism of DNA transposons in early steps. Indeed, the *rag1* and *rag2* genes have been derived from a *RAG* transposon related to *Transib* DNA transposons approx. 500–600 million years ago [73–75]. The RSSs recognized by RAG1/RAG2 might be derived from the TIRs of the ancestral transposon. The hypothesis is that, at the basis of deuterostomes, a *Transib* element originally containing only a *rag1* transposase might have captured an additional *rag2* ORF, leading to a *RAG* transposon with increased transposition activity [76]. By comparing vertebrate RAG proteins to a *RAG* transposon from the amphioxus genome that carries both *rag1*- and *rag2*-like genes [76, 77], putative key mutations in the domestication process, that impaired the transposition ability of the *rag* genes in the post-cleavage steps, have been identified [78]. This example of molecular domestication illustrates well how a specific genomic context may favor the selection and domestication of a transposable element. Indeed, for the emergence of the V(D)J recombination, the insertion of a TE with its RSS sequences into a gene encoding an immunoglobulin-domain receptor protein was probably a prerequisite to the formation of the ancestral fragmented antigen receptor gene [78].

#### TE domestication and brain development

Several retrotransposon-derived genes are implicated in vertebrate brain development, such as members of the PNMA, MART*,* SCAN and ARC gene families, that are all derived from *gag* genes of Ty3/gypsy LTR retrotransposons [68–71].

The *pnma10* gene (aka *sizn1*/*zcchc12/pnma7a*) from the PNMA gene family is involved in mouse forebrain development and mutations are associated with X-linked mental retardation in human [79]. The *pnma5* gene shows a neocortex-specific expression in primate adult brain particularly in the association areas [80]. Higher order association areas are primate-specific areas responsible for the integration of multiple inputs such as somatosensory, visuospatial, auditory and memory processes; they contribute to perception, cognition and behavior [81]. The *pnma5* gene is also present in mice but its neocortex-specific expression is not conserved. Thus, *pnma5* is thought to be one of the major genes involved in the expansion and specialization of association areas in the primate brain [80].

The protein encoded by the eutherian gene *sirh11* (aka *mart4/rtl4*), which belongs to the MART gene family, has conserved the *gag* zinc finger domain necessary for its binding to nucleic acids [70]. *Sirh11* is of crucial function for cognition [82]. Indeed, mice *sirh11* knockout mutants show impulsivity, attention and working memory defects as well as hyperactivity, suggesting a critical role in behavior. As this gene is present in eutherians only and could have conferred an essential advantage for competition by developing cognitive functions, it has been suggested to have played an important role in eutherian evolution [82].

The placental mammal gene *peg3* (*zscan24*) from the SCAN gene family has been also shown to be involved in mouse behavior [70]. This gene is paternally expressed during embryonic development and in adult brain. Its inactivation leads to growth retardation and abnormal maternal behavior for nest building, pup retrieval and crouching over pups, which can cause offspring death [83]. Moreover, mutant mothers present milk ejection defects. This phenotype has been related to a reduced number of oxytocin neurons. Growth retardation and abnormal maternal behavior are suggested to be due to impaired neuronal connectivity [83].

Finally, the *arc* tetrapod gene was shown in mice to be essential for synapse maturation and synaptic plasticity, and is involved in major neuronal processes of learning [70, 84]. *Arc* mutations have also been linked to several human disorders such as Alzheimer’s disease, Angelman neurodevelopmental disease, schizophrenia and autism among others, highlighting the crucial role of the *arc* gene in brain development and functioning [85–92]. The ARC protein has conserved structural properties similar to those of GAG proteins. Particularly, it forms capsid-like structures that transport RNA molecules across synapses and thus mediate intercellular communication between neurons [93]. Interestingly, *arc*-like genes called *darc* have been identified as duplicated copies in the genome of *Drosophila melanogaster*. Although tetrapod *arc* and *Drosophila darc* genes have been formed from Ty3/gypsy retrotransposons by independent molecular domestication events, they present similar properties of mRNA trafficking, suggesting evolutionary convergence [93, 94].

#### TE domestication and placenta development

**Fig. 2:**
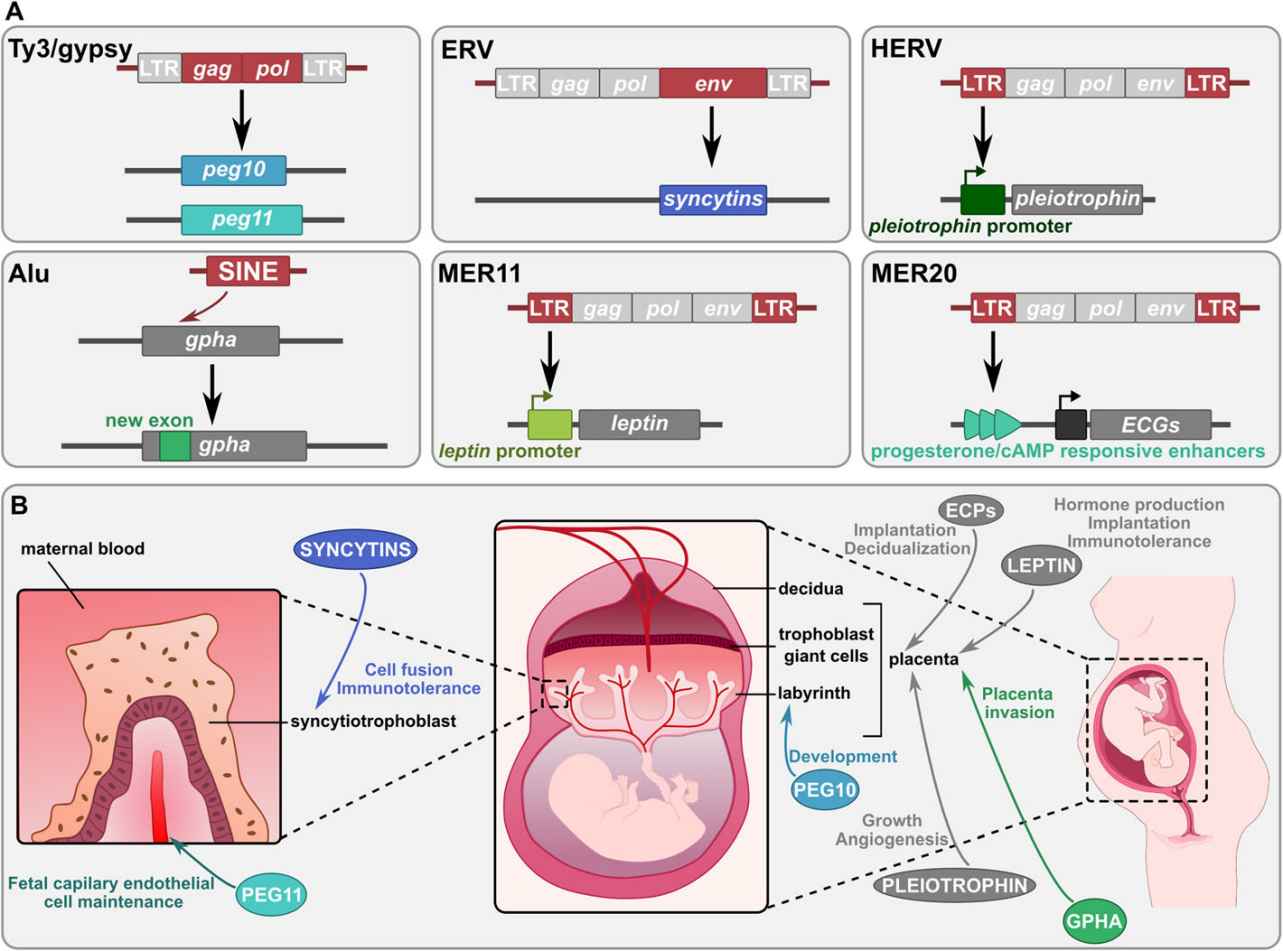
The different evolutionary contributions of TE-derived sequences to placental development. **a** Major TE co-option events in placental development. Molecular domestication of several TEs (Ty3/gypsy, ERV) has led to the formation of genes essential for placental development (*peg10*, *peg11* and *syncytins*). Alu exonization in *gpha* gene has improved placenta implantation and invasion. Co-option of TEs (ERVs) as promoter regions has led to placental regulatory circuits for several genes such as *leptin* and *pleiotrophin*. Co-option of TEs as enhancers has allowed the rewiring of placental gene networks, such as ERVs which have led to progesterone and cAMP responsive enhancers regulating placental endometrial cell gene (ECG) network. ECPs: proteins encoded by ECGs. The regions of the TE source of the co-opted sequence are represented in red in TEs and the resulting host sequences are represented in different blue/green shades. **b** Roles of the TE co-options in human placental development. The arrows illustrate the function of the proteins encoded by the genes presented in A. Baby and pregnant woman illustrations are from https://smart.servier.com

TE molecular domestication probably played crucial roles in the appearance and diversification of placenta development during mammalian evolution (Fig. 2). For instance, the *MART* genes *peg10* (aka *mart2/rtl2*) and *peg11* (aka *mart1/rtl1*) are placental genes derived from *gag* and partial *pol* sequences of Sushi Ty3/gypsy LTR retrotransposons [95, 96]. *Peg10* influences the development of the spongiotrophoblast and labyrinth layers, which are the cell layers separating the embryo from the maternal tissues of the placenta, and *peg11* maintains the fetal capillary endothelial cells. Mutation of the *sirh7* (aka *mart7/rtl7/ldoc1*) gene leads to dysregulation of placental cell differentiation and maturation linked to placental hormone overproduction [97].

*Syncytin* genes also play a central role in placenta development. They are derived from endogenous retrovirus envelope (*env*) sequences, which encode membrane proteins that allow viral fusion with the target cells necessary for infection. The SYNCYTIN proteins have kept some properties of the ancestral ENV proteins. They are able to promote cell-cell fusion, allowing trophoblast differentiation and the formation of the syncytiotrophoblast tissue, which triggers the exchange of nutrients and gases between mother and child [98–100]. Moreover, some SYNCYTIN proteins play a role in maternal immune tolerance, this being probably linked to the capacity of parental retroviruses to target and repress immune cells thanks to the immunosuppressive activity of the ENV protein [101–103]. Indeed, at least one human (SYNCYTIN-2) and one mouse SYNCYTIN (SYNCYTIN-B) show immunosuppressive activity in vivo in mouse [104].

Among placental mammals, 14 different *syncytin* genes have been identified in different lineages presenting various placenta structures characterized by different invasion levels of the uterus by trophoblast cells. The different *syncytin* genes, their expression and their properties may play a role in the placental morphological diversity observed among mammals. In sheep, the *env* gene of a very recently endogenized Jaagsiekte Sheep Retrovirus (JSRV), present at ca. 20 copies in the genome, has functions similar to those of *syncytin* domesticated genes [105]. This *env* gene indeed contributes to trophectoderm (first epithelium of the mammalian embryo) development and leads to pregnancy loss when downregulated. This might represent an example of a retrovirus gene being on the way of molecular domestication. Additionally, the human gene *suppressyn* has also been identified as an ERV env-derived gene [106]. Its protein product acts as a regulator of SYNCYTIN by binding to SYNCYTIN-1 receptor, thus inhibiting SYNCYTIN-1-mediated cell fusion.

Interestingly, *syncytin* genes in different lineages are not orthologous and have been formed by independent events of molecular domestication of ERV envelope genes, testifying for a fascinating case of convergent evolution. This underlines how TEs can represent (almost) ready-to-use molecular material that can be repurposed independently several times during the evolution of different lineages. In addition, it has been recently demonstrated that ERV *env* sequence captures are not specific of eutherian mammals, since other *syncytin* genes of independent origins have been found in marsupials and even in some viviparous lizards [107, 108].

Mammalian placenta evolution through the molecular domestication of several different retrotransposon and retrovirus genes has been proposed to follow a “baton pass” mechanism [109]. First, the early birth and high conservation of the three LTR retrotransposon-derived genes *peg10*, *peg11* and *sirh7* among mammals suggest that they could be at the origin of the primitive placenta at the base of placental mammals. Subsequently, an ancestral gene responsible for cell fusion may have been substituted by *syncytin* gene(s), which might have then replaced one another, ensuring or even improving the function and the performance of the previous *syncytin* gene, and allowing placenta morphological innovations [109, 110].

Placenta appears thus to be the place of multiple events of TE co-option. Some studies suggest that these domestications may have been facilitated by the hypomethylation of DNA in placenta compared to other tissues, allowing higher TE expression and subsequent easier TE recruitment [111, 112].

#### TE domestication and the diverse roles of the ZBED family

The ZBED gene family derives from *hAT* DNA transposons, and more precisely from the BED zinc finger domain of their transposase, which is involved in DNA binding [113]. This gene family is implicated in various aspects of tissue or organ development in vertebrates. For example, the mammalian ZBED3 binds to the AXIN protein to form a complex that regulates the Wnt/β-catenin signaling pathway, which is essential for embryogenesis and carcinogenesis [114]. In addition to the BED domain, *zbed1*, *zbed4* and *zbed6* also kept the DDE catalytic domain of the ancestral TE transposase, which contains an ⍺-helical domain and a dimerization domain. Present in bony vertebrates, *zbed4* is proposed to be involved in retinal morphogenesis and in the functioning of Müller retinal glial cells by activating the transcription of genes expressed in Müller cells or by regulating their nuclear hormone receptors [115]. The placental mammal gene *zbed6* encodes a transcription factor essential for muscle development. A single nucleotide (nt) mutation in an *igf2* intronic sequence prevents the repression of this gene by ZBED6, leading to an increase in muscle growth and heart size and to a decrease in fat deposition [116]. ChIP-sequencing experiments have revealed about 1200 additional putative genes targeted by ZBED6, with particular enrichment in genes involved in development, cell differentiation, morphogenesis, neurogenesis, cell-cell signaling and muscle development. Finally, the vertebrate gene *zbed1* is implicated in cell proliferation by regulating several ribosomal protein genes [117, 118].

## TEs as a source of new non-coding RNA genes

### TE-derived small non-coding RNAs

**Fig. 3:**
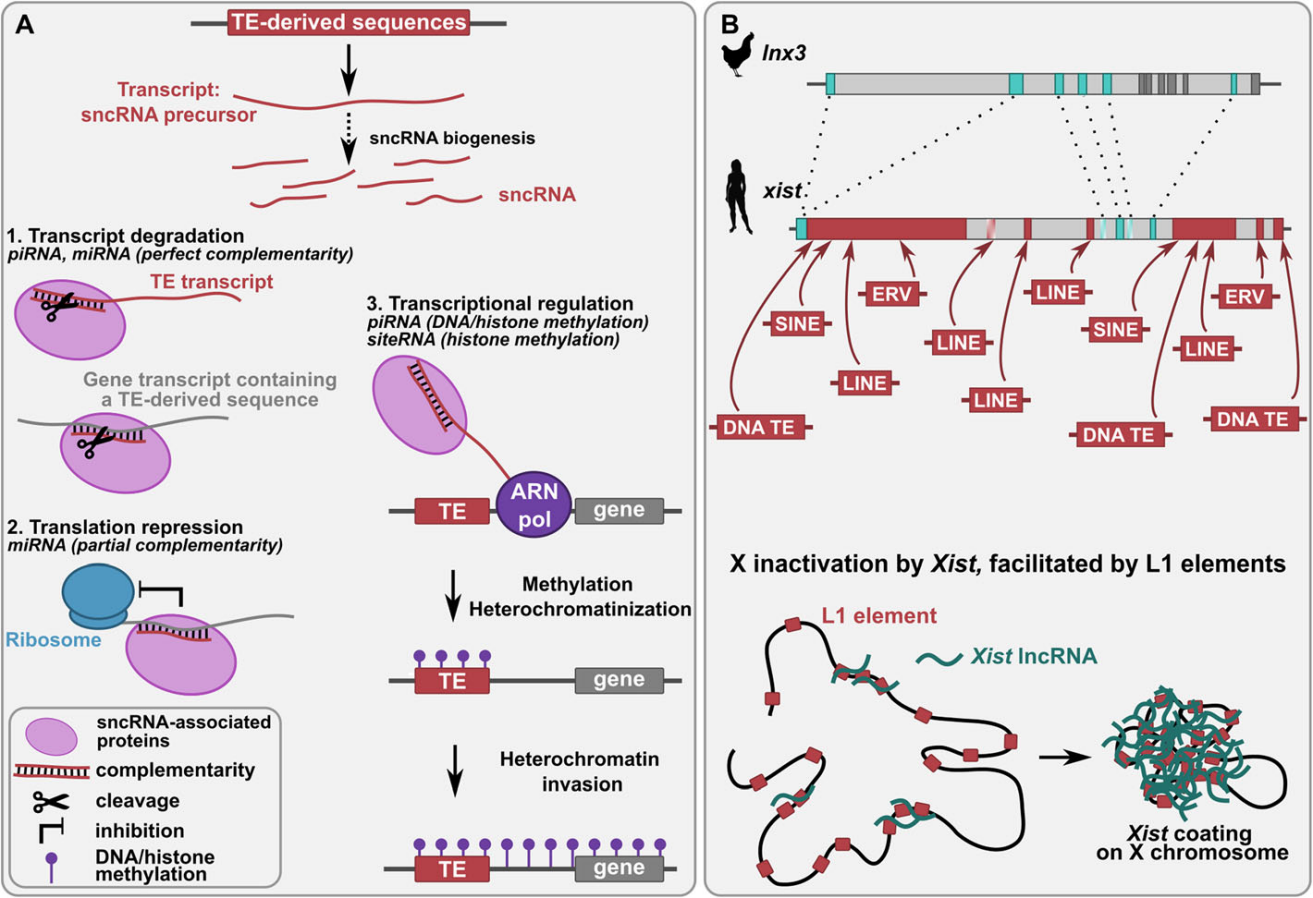
Functions of TE-derived non-coding RNAs. **a** Mechanisms of action of TE-derived small non-coding RNAs (sncRNAs) through sequence complementarity. TE-derived sncRNAs are formed by fragmentation of TE-derived transcripts [122, 294], siRNAs being generated through the cleavage of the successive precursors pri-miRNAs and pre-miRNAs [122]. TE-derived sncRNAs, associated to proteins (RNA-induced silencing complex for miRNAs [122], PIWI proteins for piRNAs [150]) form double-stranded RNAs with complementarity to some RNAs of the host transcriptome, this leading to the cleavage of RNAs (1) and to the inhibition of translation (2). sncRNAs also mediates the heterochromatinization of TEs to silence them after the recruitment of DNA and histone methyltransferases (3). This heterochromatinization can spread to neighboring regions, altering their expression. **b** Evolution and function of the *xist* gene. Top: the human *xist* lncRNA gene has been formed after ancient insertions of several TEs (red boxes) into the ancestral protein-coding *lnx3* gene, which is still present in chicken. *lnx3* blue boxes represent the exons homologous to *xist* exons and dark grey boxes other exons. *Xist* shaded boxes represent human pseudo-exons (intronic regions in human but exonic in other species). Red arrows indicate TE and *xist* exon homology. Bottom: *Xist* lncRNAs coat the X chromosome, leading to X chromosome inactivation, which is facilitated by LINE-1 elements present on the chromosome [190, 191]. Silhouette images from http://phylopic.org

TE sequences can be a source of small non-coding RNAs (sncRNAs) (Fig. 1c). Several studies have shown that some sncRNAs can derive from TEs, such as microRNAs (miRNAs) [119] and Piwi-interacting RNAs (piRNAs) [120]. These sncRNAs generally constitute TE silencing factors, but they have also shown abilities to regulate host gene expression by sequence complementarity through mRNA degradation and translation inhibition (Fig. 3a). sncRNAs can also induce DNA methylation of the loci close to the nascent mRNA their target. This can induce heterochromatinization, which can spread in the targeted genomic region and thus can potentially lead to the transcriptional repression of neighboring genes (Fig. 3a) [121].

#### TE-derived miRNAs

TEs have contributed to the formation of miRNAs that play important roles in vertebrate developmental processes such as cell differentiation, maternal mRNA clearance and brain development [122–128]. miRNAs are sncRNAs with an average of 22 nt in length that are generated after the cleavage of 70–90 nt precursor miRNAs (pre-miRNAs), which are themselves produced by the cleavage of primary miRNA (pri-miRNA) transcripts [122]. Through complementary binding, miRNAs regulate mRNA degradation and translation. In the case of perfect sequence complementarity between miRNA and mRNA, the mRNA molecule will undergo endonucleolytic cleavage. Partial complementarity will lead to translational repression.

About 20% of human miRNAs are derived from TEs [119]. This proportion seems to be lower in other vertebrates, from 0% in the Western clawed frog to 15% in rhesus macaque and mouse [119]. In human and globally in other vertebrate species, DNA transposons make the highest contribution to miRNAs, followed by non-LTRs (LINEs and SINEs) and LTR elements; proportions that generally do not reflect the relative amount of the different types of TEs in species genomes [124, 126].

TE-derived miRNAs appear to be less conserved than non-TE-derived miRNAs, suggesting that they could constitute more lineage-specific regulators allowing the emergence of potential new phenotypes [124]. TE sequences present in the untranslated regions (UTRs) of genes constitute main targets for TE-derived miRNAs, in particular LINE1-, Alu*-* and MIR-derived sequences in mammals [128, 129]. The expansion of TE families such as Alu elements in primates or B1 SINEs in rodents has led to lineage-specific miRNA target sites and thus to lineage-specific regulatory potential [128].

Among the TE-derived miRNAs with a role in processes linked to development in vertebrates, miR-587, a miRNA derived from a MER element (MEdium Reiteration frequency, non-autonomous DNA transposon), has been shown to be implicated in cell cycle progression in human by regulating the *tgfbr2* and *smad4* genes [130]. Another miRNA, miR-122, is involved in liver metabolic functions and is essential for the differentiation of hepatoblasts, the fetal precursor of liver cells, in zebrafish [131, 132].

Several miRNAs are involved in myeloid regulation in mouse and human. As an example, miR-652, which is derived from a MER element, is specific of myeloid lineage cells and is supposed to regulate cell identity by targeting cell type-specific regulatory proteins [133–136]. miR-935, miR-720, miR-422 and miR-378, which have been formed from different types of TEs, are all specific of one particular myeloid cell type: mucosal mast cells for miR-935, neutrophils for miR-720 and monocytes for miR-422 and miR-378. However, their precise roles remain to be elucidated. miR-378 has also been shown to be involved in myoblast differentiation and has a pro-angiogenic and possible anti-inflammatory effect during skeletal vascularization in mice [137].

The mammalian miR-340 and miR-374, respectively derived from a *Mariner* DNA transposon and a L2 non-LTR retrotransposon, are regulators of the microtubule-associated MIDI protein, an E3 ubiquitin ligase that is an activator of the mammalian Target Of Rapamycin (mTOR) in a signaling pathway essential for cell proliferation, growth and mobility, and protein biosynthesis among others [138–140]. MIDI mutations cause the Opitz BBB/G syndrome, characterized by ventral midline malformations, with defects in heart, palate and brain structure, and hypertelorism and hypospadias [141]. In rodents, miR-374 has been shown to regulate the differentiation of myoblasts [142] and chondrocytes [143], and plays a role in retinal ganglion cell development [144]. This miRNA is also involved in primary porcine adipocyte differentiation [145] and in the production of goat hair [146].

The miR-513 subfamily, derived from a MER element, is composed of several miRNAs resulting from successive duplications in primates [147]. miR-513b regulates at both mRNA and protein levels the DR1 (down-regulator of transcription 1) protein, which is a phosphoprotein associated with TBP (TATA box-binding protein) that represses transcription. As TBP is important for spermatogenesis in mammals, miR-513b might participate in male sexual maturation by regulating DR1 [148].

#### TE-derived piRNAs

piRNAs are 24–31 nt long sncRNAs that together with PIWI proteins (such as MILI, MIWI and MIWI2) form complexes implicated in TE repression in the germ line and in gene regulation [149–152]. piRNA/protein complexes recognize mRNAs by complementarity with the piRNA sequence. The target mRNA is then cleaved, leading to its degradation and to the formation of secondary piRNAs that can in turn target additional complementary mRNAs. These complexes also induce DNA methylation of the regulatory regions of the mRNA they target [149, 153]. piRNA targeting is not restricted to identical sequences, this relaxed specificity increasing the number of possible targets [154]. piRNAs are major actors in TE inactivation and can thus prevent the deleterious transposition of TEs in germ cells [155]. Several studies have demonstrated the evolutionary conservation of the piRNA pathway, suggesting important functions particularly during development [156].

The origin of piRNAs is not always well characterized. piRNAs can either derive from remnant TE sequences (i.e. ancient insertions of TEs in genomic piRNA clusters) or from single insertions of active TEs [120]. TE insertion into genes can therefore represent a way to regulate genes through their targeting by TE-derived piRNAs [157]. piRNAs might also be formed from non-TE sequences, but a very ancient TE origin not detectable at the sequence level due to divergence can often not be excluded. piRNA clusters can evolve rapidly, allowing interesting adaptation ability [158].

In mammals two populations of piRNAs are of particular importance during spermatogenesis: pre-pachytene and pachytene piRNAs, which correspond to piRNAs expressed at two distinct stages of male germ cell development [151, 159, 160]. Pre-pachytene piRNAs are expressed during early stages of spermatogenesis and in fetal and perinatal male germ cells, and are associated with the MILI and MIWI2 proteins [149, 161]. Pachytene piRNAs are produced in pachytene spermatocytes and post-meiotic spermatids, and form complexes with the MILI and MIWI proteins [160, 162]. Knockout of the proteins associated with both types of piRNAs causes male infertility [151, 159].

Most pre-pachytene piRNAs have been shown to derive from TE sequences, with SINEs (49%), LINEs (16%) and LTR elements (34%) being the main contributors in mouse [149]. They are directly involved in the de novo DNA methylation of TE sequences but also of genes and other non-TE sequences, probably through their binding to genomic DNA or nascent transcripts [153, 160, 161, 163]. Pachytene piRNAs are essential for the degradation of complementary mRNA in spermatids and maternal mRNA in early embryos, regulations that contribute to correct germ cell and embryo development. Mouse pachytene piRNAs are formed from about 3000 genomic clusters [164]; most of them target retrotransposon sequences, and more particularly SINE elements [160]. Pachytene piRNAs, some of them derived from TEs, have also been identified in bovine, macaque and human female germline and have been suggested to be involved in oogenesis and early embryogenesis [165].

#### TE-derived siteRNAs

A new class of sncRNAs called siteRNAs (for small intronic transposable element RNAs) has been defined in the frog *Xenopus tropicalis* [166]. These sncRNAs are 23–29 nt in length and derived from TE sequences inserted in introns of protein-coding genes. They have the ability to participate in the transcriptional silencing of the genes from which they originate by recruiting repressive histone marks (Fig. 3a). Thus, by targeting TE sequences, this TE silencing mechanism acts on regions flanking TE insertions.

### TE–derived long non-coding RNAs

Long non-coding RNAs (lncRNAs) are non-coding RNAs longer than 200 nt in length. They include long intergenic non-coding RNAs (lincRNAs) that do not overlap with protein coding-genes and make up more than half of lncRNAs in human [167]. LncRNAs can act as chromatin, transcription and post-transcription regulators through the recruitment of transcription factors and chromatin-remodeling complexes, as well as through interactions with the RNA polymerase machinery, splicing factors and mRNAs by sequence complementarity [168]. LncRNAs and more particularly lincRNAs have been shown to be implicated in many cellular [169, 170], epigenetic [171–174] and developmental processes [175], such as transcriptional silencing, cellular reprogramming and X chromosome inactivation. LncRNAs are also involved in erythroid, myeloid and lymphoid development (reviewed in [176]). They are highly expressed during central nervous system development and more particularly during neuronal and retinal differentiation, in a very time- and region-specific manner (reviewed in [177]). They are often associated to nervous system disorders.

In vertebrates, most lncRNAs in each species are lineage-specific, indicating their rapid evolutionary turnover [178, 179]. The majority of lncRNAs are thus young, and new lncRNAs are formed at a very high rate compared to protein-coding genes (ca. 100 new genes per million years in primates and rodents) [178]. lncRNA expression also seems to evolve faster than that of protein-coding genes [178, 180–182]. However, a thousand human lncRNAs are likely to have conserved functions across mammals, and hundreds beyond mammals [179].

A major part of vertebrate l ncRNAs and lincRNAs contains TE-derived sequences (Fig. 1c), the estimations ranging from 50 to over 80% depending on the study and the species considered [183–186]. Within lincRNAs, which experience the same maturation steps as pre-mRNAs of protein-coding genes but are frequently poorly spliced [187], TE-derived sequences are preferentially found in introns and then in exons and promoters in mammals [185]. In a study focusing on human and mouse, the contribution of the different TE families to lncRNAs was found to reflect globally the amount of each family in the genome, except for a depletion of LINEs in lncRNA exons and promoters [185]. Within a species, the contribution of TE-derived sequences in terms of coverage can be very variable depending on the lncRNA considered. In human, TE coverage between different lncRNAs ranges from 0 to 95%, with half of lncRNAs being covered by more than 20% of TE-derived sequences [184]. Some TE-derived sequences are of functional importance by allowing notably the formation of RNA-, DNA- or protein-binding domains [188]. In human, LINE2 and MIR elements drive the nuclear enrichment of lncRNAs that allows them to modulate gene expression [186].

Even in conserved lncRNAs, sequence conservation is generally unequal along the lncRNA molecules, with small patches of high conservation separated by less constrained sequences [179]. This is consistent with a high rate of exon gain/loss and exon/intron structure modification [172]. Such a pattern might be indicative of a tolerance for sequence evolution by TE acquisition in lncRNA genes. TEs are therefore likely to be major actors of the rapid evolutionary turnover of the lncRNA repertoire in species, since they can be source of novel transcription initiation, splicing, polyadenylation and regulatory sites, as well as of new exonic sequences.

#### TE-derived lncRNAs in X chromosome inactivation

One best studied example of TE-containing lncRNA is *Xist*, which is involved in X-chromosome inactivation in females of eutherian mammals [189]. Inactivation of one X chromosome is essential for the dosage compensation of X-linked genes in females (XX) compared to males (XY), which have only one X chromosome. Six of the ten exons of the *Xist* lncRNA show similarities to SINEs, LINEs or DNA transposons [172] (Fig. 3b). Some of these TEs, particularly LINEs, are essential for *Xist* addressing and for inactivation of the X chromosome in mouse [190, 191]. *Xist* lncRNA colocalizes with LINE elements and probably binds to these sequences, which cover a large part of the X chromosome [192]. These interactions are thought to be essential for the establishment of X chromosome inactivation.

The primate-specific *Xact* lncRNA is rich in repetitive elements, particularly in LTR-derived sequences [193]. *Xact* coats the active X chromosome and has been proposed to act as a transient *Xist* antagonist inhibiting inactivation. A *Xact* enhancer is derived from an ERV and is responsible for *Xact* expression in human pluripotent cells [193].

#### TE-derived lncRNAs in embryonic stem cells

Some TE-derived lncRNAs present a conserved expression in induced pluripotent stem cells of different primate species, suggesting an important function that remains to be uncovered [194]. Several lncRNAs are involved in maintaining embryonic stem cell pluripotency, with a particular influence of LTR-derived sequences [195–197]. For example, a human ERV-lncRNA has a domain that can recruit RNA-binding proteins, pluripotency factors and histone modifiers [197]. Human ERVs can form a hundred of lncRNAs that are specific for human pluripotent stem cells and ensure their cell identity and pluripotency [169, 183, 196, 198]. LINE1 RNAs can act as lncRNAs and chromatin regulators, and are involved in mouse embryonic stem cell self-renewal and preimplantation embryo development. These effects occur via the activation of rRNA expression and the repression, through the recruitment of Nucleolin and Kap1/Trim28, of the *dux* developmental gene, which encodes a transcription factor activating a program specific to 2-cell embryos [199, 200].

#### TE-derived lncRNAs in brain development

A recently described class of lncRNAs, called SINEUPs, up-regulates translation through an embedded inverted SINE element that forms a short hairpin [201, 202]. This hairpin has been shown to be essential for the up-regulation function of SINEUP lncRNAs and serves as a recognition motif for the RNA-binding protein ILF3 (IL enhancer-binding Factor 3) [203]. The first representative member of this family, which was described in mouse, is responsible for the translational regulation of the ubiquitin carboxy-terminal hydrolase L1 (*uchl1/PARK5*), which is essential for brain function and particularly for neuron maintenance [201, 204, 205]. This SINEUP lncRNA, which carries a SINEB2 element, is antisense to *uchl1*. Another antisense SINEUP lncRNA, isolated from human brain, contains a free right Alu monomer element and increases the translation of the gene expressing the phosphatase 1 regulatory subunit 12A (PPP1R12A) [206]. PPP1R12A presents human pathogenic variants that have been associated with a congenital malformation syndrome affecting brain embryogenesis [207] and is involved in the development of the central nervous system in zebrafish [208]. More than 100 potential additional antisense SINEUP lncRNAs expressed in human brain have been identified [206], revealing other candidates for SINEUP-regulated genes involved in brain development and functioning. Interestingly, analysis of these genes indicates that different SINE elements can potentially function as effector domains in SINEUP lncRNAs [206].

Non-SINEUP examples of lncRNAs involved in brain development include the vertebrate lincRNA *cyrano,* the polyA signals of which are embedded in different TEs (LTR, SINE or LINE) depending on the transcript [184]. *Cyrano* has been shown to be essential for proper embryonic development and neurodevelopment in zebrafish [184, 209, 210]. The lincRNA *megamind* is implicated in brain morphogenesis and eye development in vertebrates. Its transcription starting site is located in a L3 LINE element in mammals, but it is not known if *megamind* uses the original promoter of the retrotransposon for its transcription [184, 209].

## TE-derived sequences as a source of new regulatory elements

### TE-derived sequences as new developmental cis-regulatory elements

Many studies have established the capacity of TEs to be bound by transcription factors, a property that has been repeatedly used in host genomes to form new gene regulatory sequences and networks [27, 211] (Fig. 1d/e). For example, the ESR1, TP53, POU5F1, SOX2 or CTCF (CCCTC-binding factor) proteins are able to bind to TE sequences [211]. This ability has been shown to be essential for mammalian evolution since it can occasionally mediate the rapid expansion of transcription factor (TF) binding sites carried by the TEs and consequently the evolution of regulatory networks. As assessed by ChIP-seq technology, as much as 20% of transcription factor binding sites (TFBS) in human and mouse genomes are embedded in TEs, and this can range from 2 to 40% depending on the TF [212]. TE-derived regulatory sequences are often associated with active chromatin regions that are species-specific, suggesting their major involvement in the evolution of species-specific regulations [212]. A recent genome-wide analysis characterized human molecular pathways associated with retrotransposon-derived TFBS [213]. Olfaction, color vision, fertilization, cellular immune response, amino and fatty acids metabolism and detoxification were found to be particularly enriched for retrotransposon-derived gene regulation, i.e. mainly pathways with strong lineage/species specificity. The analysis of the association between TEs and active/repressed chromatin marks across 24 human tissues showed that SINEs and DNA transposons are enriched in globally active regions, while LTRs show a more tissue-specific enrichment [214]. Moreover, TEs enriched in tissue-specific regulatory regions present binding sites for tissue-specific TFs, and their expression correlates with the tissue-specific expression of neighboring genes. This indicates that TEs can serve as a major source for regulatory sequence turnover in a tissue-specific manner, as observed in human and mouse [214, 215].

In addition to enhancers and silencers, TEs can form new gene promoters. As much as 11 and 16% of RNA polymerase II binding sites have been estimated to be derived from TEs in mouse and human genomes respectively [212]. In mouse and primates, multiple RNA polymerase II promoters have been formed from SINEs, which are different from the polymerase III promoters that are classically used by these elements [216, 217]. LTR elements are also a source of new gene promoters [218], for instance in embryonic developmental genes (see below).

The *wnt5a* enhancer illustrates well the potential of TE-derived sequences in the evolution of developmental programs [219]. The *wnt5a* gene is a secreted signaling protein important for vertebrate embryogenesis [220]. This enhancer, which is essential for the morphological evolution of the mammalian secondary palate, has been formed by a combination of different TE sequences (AmnSINE1, X6b_DNA and MER117). Each TE sequence contributed to different tissue-specific enhancer activities, cooperatively allowing an expression pattern compatible with the formation of the whole secondary palate. This example illustrates how a combination of TE-derived enhancers can generate the fine-tuned and complex diversification of developmental enhancers during evolution.

#### TE-derived regulatory sequences in early embryogenesis

Many TEs are involved in the expression landscape of early mouse embryos [221]. In particular, LTR elements have a strong impact on the expression of neighboring genes at earliest stages, probably through the recruitment of homeobox factors. SINE elements also induce the expression of neighboring genes during zygotic genome activation and in embryonic stem cells [221]. TEs and particularly ERVs have given rise to hundreds of thousands of primate-specific regulatory elements, and among these sequences thousands are activated specifically in embryonic cells concomitantly with neighboring genes [222]. TEs can be major actors in the formation and evolution of specific developmental regulatory networks, as demonstrated for OCT4 and NANOG, two transcription factors essential for early embryogenesis and embryonic stem cell pluripotency in mammals. A high proportion of the binding sites of these proteins are indeed derived from TEs, in particular ERV elements (21% in human and 7% in mouse for OCT4, 17% in both human and mouse for NANOG) [223].

The evolvability that TEs can confer to vertebrate developmental regulatory networks is well illustrated by mammalian embryonic stem cells. The regulatory networks of these cells are plastic, and this plasticity is at least partially due to the species-specific co-option of TEs as enhancers and promoters [223]. The potency of mouse embryonic stem cell depends on the promoter activity of MERV (murine ERV) LTRs [224]. MERV LTRs can act as promoters for two-cell stage (2C) genes, i.e. genes normally expressed in early developmental stages and repressed thereafter, this modifying cell fate. Similar results were obtained for human ERVs (HERV) [225]. HERV/LTRs can be grouped depending on the TFBS they carry. Four main patterns of TFBS were identified: binding sites for pluripotent TFs (such as SOX2, POU5F1 and NANOG), for embryonic endoderm/mesendoderm TFs (such as GATA4/6, SOX17 and FOXA1/2), for hematopoietic TFs (such as SPI1/PU1, GATA1/2 and TAL1) and for CTCF.

In vertebrates, TE-derived sequences can be targeted by Kruppel-associated box zinc finger proteins (KRAB-ZFPs) [226]. KRAB-ZFPs are early embryonic controllers that mediate the methylation of histones and DNA, inducing the repression of targeted TEs and TE-derived sequences. This can impact the expression of neighboring genes and control regulatory networks acting during early development. Consequently, it has been proposed that the expansion of the KRAB-ZFP family results not only from the necessity of controlling TEs but could be an innovative way to build new regulatory networks through TE exaptation and controlling [226].

#### TE-derived regulatory sequences in brain development

SINEs are of particular importance for mammalian brain development. For instance, two SINE insertions recruited as enhancers in a mammalian common ancestor are involved in brain development [227]. The fibroblast growth factor 8 (*fgf8*) gene encodes a factor required for embryonic development, morphogenesis and particularly for normal brain, eye, ear and limb development. The first SINE insertion controls the expression of the *fgf8* gene in the diencephalon and the hypothalamus. This allows the mammalian-specific patterning of the forebrain, which is the most complex region of the vertebrate central nervous system, implicated in diverse functions such as body temperature homeostasis, sleeping, eating and reproductive function regulation, as well as in the display of emotions. The second SINE insertion regulates the *satb2* gene, which is a DNA binding protein involved in chromatin remodeling and essential for telencephalon functioning [228, 229].

An insertion of the MER130 SINE is involved in the development of the neocortex, a mammalian-specific structure responsible for the implementation of cognitive, emotive and perceptive functions [230]. This TE works as an enhancer of critical neocortical genes. A tetrapod LF-SINE-derived enhancer controls the *islet-1* (*isl1)* gene, which encodes a transcription factor essential for tetrapod brain development, particularly for motor and sensory neuron differentiation [231, 232].

Interestingly, a new regulatory function has been identified for SINEs in mouse neurons [233]. In neurons, synaptic activity influences gene expression through epigenetic modifications and the recruitment of regulatory proteins. SINE sequences located close to activity-regulated genes act as regulators for their expression. In response to neuron depolarization, these SINE sequences are acetylated, inducing the binding of the transcription factor TFIIIC. TFIIIC recruitment allows activity-dependent transcription, the relocation of inducible genes to transcription factories (i.e. specific nuclear foci where stimulation-responsive genes are expressed), as well as dendritogenesis [233]. In this context, the binding of TFIIIC to SINEs mediates the coordination of the nuclear architecture, allowing activity-dependent gene expression.

Finally, TE-derived sequences can be involved in neural gene *cis-*regulation through epigenetic modifications [234]. Indeed, TEs can be silenced by DNA methylation, which prevents transposition. This silencing can affect surrounding sequences, altering neighboring gene expression. Hypomethylated TE-derived sequences are associated with active tissue-specific enhancer marks. This allows these sequences to gain active functions in tissue-specific gene expression [234]. This mechanism appears to be essential for the development of brain and specifically of neurons in human. For instance, the hypomethylation of the *UCON29* DNA transposon and the LF-SINE retroelement, which occurs only in fetal brain, allows the transcriptional activation of several neuron and telencephalon developmental genes specific to human [234].

#### TE-derived regulatory sequences in liver development

Liver developmental evolution is also linked to TE exaptation. A recent analysis of liver *cis-*regulatory elements evolution within primates distinguished two types of sequences: those conserved within primates, which represent 63% of liver *cis-*regulatory elements, and those that are not conserved, which correspond to newly evolved regulatory sequences mostly derived from TEs [235]. The majority of these sequences arose from TEs having recently transposed, particularly LTR retroelements and SINEs. Moreover, newly evolved *cis-*regulatory elements are species-specific and are associated with the species-specific binding of transcription factors involved in liver functions. They are also associated with immune- and neuro-developmental functions.

#### TE-derived regulatory sequences in sexual development and gametogenesis

Several examples illustrate how TEs can be involved in the control and evolution of sexual development in vertebrates. In the medaka fish *Oryzias latipes*, a DNA transposon called *Izanagi* controls the expression of the master gene regulator of male development *dmrt1bY* [236]. *dmrt1bY*, located on the medaka Y chromosome, appeared through the duplication of the autosomal *dmrt1* gene, a male gene acting downstream in the sex determination cascade. The co-option of the *Izanagi* TE-derived sequence allowed *dmrt1bY*, by inducting a new regulation, to take the lead of the sex-determining cascade of the medaka.

Estrogen receptor ⍺, FoxA1, GATA3 and AP2 are crucial regulators of mammary gland development. The expansion of retrotransposons in mammals has given rise to thousands of binding sites for these regulators [237]. Such a spreading particularly resulted from the expansion in two phases of L2/MIR elements in a eutherian ancestor, and of ERV1 elements in simians and rodents. These retrotransposon-derived sequences act as enhancers and their recruitment allowed the establishment of the gene network of the mammary gland regulators, allowing its morphological innovation.

LTR elements are involved in oogenesis in mammals [238]. They can form enhancers, promoters and first exon sequences of host genes and thus lead to a synchronized and developmentally regulated expression of genes. More than 800 LTR elements, mainly from the ORR1, MT, MT2 and MLT families, gave rise to promoters and first exons in mouse genes expressed in oocytes and early embryos [239]. These elements can activate the transcription of their neighboring genes during the oocyte-to-embryo transition. For example, an MTC LTR element is at the origin of the oocyte-specific high-activity isoform of Dicer (protein involved in sncRNAs biogenesis) in mouse. The deletion of this MTC element causes meiosis spindle defects and an increase of endo-siRNA target levels, and finally leads to female sterility [240]. LTR sequences are also involved in vertebrate spermatogenesis by acting as tissue-specific promoters of protein-coding and lncRNA genes [241].

#### TE-derived regulatory sequences in placenta development

TE sequences have been repeatedly selected, often in a lineage-specific manner, as new regulatory elements for mammalian placental development, sometimes in association with new TE-derived genes (Fig. 2). It has been shown for example that the ERV-derived *syncytin-1* is regulated by a TE-related sequence in human. Indeed, an LTR promoter combined to an adjacent cellular enhancer is responsible for the high expression of *syncytin-1* in placenta [242].

Ancient TEs have been key actors of the establishment of the decidualization, i.e. the differentiation of endometrial stromal fibroblasts into decidual stromal cells in response to different signals such as progesterone [243]. Decidualization is a key step of pregnancy establishment and maintenance, because it allows maternal-fetal communication and maternal immunotolerance. Strikingly, the exaptation of thousands of TEs has allowed the endometrial expression of numerous genes that were ancestrally expressed in other tissues [243]. Rewiring of these genes was responsible for the apparition of new functions such as immune response regulation and maternal-fetal signaling. The rewiring capacity of TEs, considered to be a major mechanism at the origin of pregnancy, was explained by the fact that they bring enhancers responsive to progesterone and cAMP, as well as TFBSs for master transcriptional regulators responsible for endometrial stromal cell-type identity [243, 244]. This was particularly suggested for the eutherian-specific MER20 DNA transposon, which has played a major role in the rewiring of the placental endometrial cell gene network [244].

More specifically, LTR promoters allow the trophoblast-specific expression of placental genes such as *pleiotrophin* and *leptin* in human [245, 246]. Pleiotrophin is a growth factor with mitogenic, growth promoting and angiogenic activities [247]. Leptin is a hormone essential for reproductive function. It is necessary for gonadotrophin hormone production, placentation and embryo implantation, and acts as an immunomodulator [248]. Another ERV (MER21A) gave rise to a placenta-specific promoter for the *cyp19* gene in primates [249, 250]. *Cyp19* encodes the aromatase P450 essential for estrogen synthesis; mutations and expression alterations of this gene are associated with reproduction abnormalities such as infertility and ovulation failure [251]. Thus, this ERV co-option is assumed to be of major importance for estrogen regulation during primate pregnancy. Finally, the promoter sequence of a LINE family is used to drive the placenta-specific expression of lncRNAs in human [252].

TE-derived enhancers are of peculiar importance for the regulation of the *prolactin* (*prl*) gene [253, 254]. PRL is a hormone involved in lactation as well as in the regulation of immune system, metabolism, pancreatic development and placental implantation during eutherian pregnancy. Its expression is promoted by MER20/MER39 ERV, MER77 ERV and LINE-1-derived enhancers in human, mice and elephant respectively, these regulatory sequences being progesterone- and cAMP-responsive [255]. TEs are also main contributors of the trophoblast stem cell (TSC) regulatory network, ERV retroelements forming hundreds of mouse-specific enhancers that can recruit TSC-determining factors such as CDX2, EOMES and ELF5 [256].

A two-step model has been proposed to explain the role of TEs in the evolution of mammalian placenta [112]. The first step consists in an ancestral acquisition of ERV-derived regulatory sequences responsible for the recruitment of genes to build a new network controlling placenta development, this allowing the rise of an ancestral form of placenta. Then, a relaxed repression of ERVs in trophoblast cells and the capture and replacement of *syncytin* genes facilitated the lineage-specific divergence of this network, allowing the developmental diversification of mammalian placentas that we observe today. The transient state of the placenta during life cycle may have favored its evolution and multiple TE co-options, by limiting harmful TE mutagenic activity [112].

### TE-derived sequences involved in chromosomal architecture and chromatin organization

Chromosome 3D organization is essential for multiple processes such as replication, chromosome segregation during meiosis and mitosis, transcription and long-distance gene regulation, which are indispensable to ensure proper organism development [257]. Alterations in this genome organization can lead to developmental disorders such as limb syndromes and neurodevelopmental disorders (ex. Hutchinson–Gilford progeria and Warsaw Breakage syndromes), as well as to psychiatric disorders [258–260].

It has been demonstrated that TE-derived sequences can be involved in chromosome architecture (Fig. 1f). They can provide insulator regions, which can partition the genome into topologically associated domains (TADs) and smaller chromosomal loops, and can hinder interactions between adjacent enhancers and promoters [261, 262]. CTCF, a zinc finger protein that is the only insulator protein identified so far in vertebrates, is responsible for the proper separation of different chromatin domains [263]. TEs such as SINE B2, HERV and MER20 DNA transposons can be bound by CTCF [225, 244]. Strikingly, 40% of CTCF binding sites are located in TEs in mouse genome [212]. Accordingly, it has been shown that 12–18% of human loops and 15–27% of mouse loops are indeed associated with repetitive element-derived CTCF anchor sites, the great majority of them being TEs [264].

Looking at multiple mammalian genomes, several conserved ancient retrotransposon sequences surround CTCF-binding sites, suggesting that TE expansion tens of million years ago may have given rise to mammalian and probably vertebrate conserved CTCF insulator regions [265]. On the other hand, CTCF-binding TEs have mainly enabled the species-specific expansion and diversification of CTCF binding regions in vertebrates, which are otherwise generally very constrained [265, 266]. This is likely to promote gene expression diversification between cells and between species [267], as proposed for SINE invasion in dog, rodent and opossum genomes [265]. Accordingly, multiple TEs can form chromatin loop anchors in a species-specific manner: in human, LTR, LINE and DNA transposons mostly contribute to CTCF anchors, while in the mouse SINEs, and particularly the B2 SINE family, are the main contributors [264]. Interestingly, the ChAHP complex (a protein complex constituted by the chromatin remodeler CHD4, the transcription factor ADNP and heterochromatin-binding protein HP1) binds at younger, less divergent SINE B2 elements and competes with CTCF for binding, buffering the genome architecture rewiring, associated with SINE B2 expansion in mice [268]. Most TE-derived CTCF anchors are cell-type specific, showing the potential of TEs to influence cell-type specific expression programs. TE-derived anchors are also hypomethylated, consistent with the fact that CTCF only binds unmethylated DNA.

In hominid pluripotent stem cells, HERV-H elements have been shown to be able to form TADs [269]. Deletion of HERV-H sequences induces the loss of their corresponding TADs and leads to a reduction of transcription of upstream genes. Conversely, the insertion of novel HERV-H copies is able to form new TADs. Repression of HERV-H transcription induces TAD loss, suggesting an importance of HERV-H expression in TAD formation [269]. In the human genome, insulators can also arise from MIR retrotransposons, but in a CTCF-independent manner [270]. They are characterized by an RNA Pol III transcription and various histone modifications that can directly impact chromosomal organization.

In mouse, the SINE B2 repeat has been linked to organogenesis through its dynamic insulator activity [271]. Bidirectional transcripts of a SINE B2-derived sequence located upstream of the murine *growth hormone* gene (*gh*) are synthetized using both Pol II and Pol III promoters. These transcripts act as boundary elements by perturbing chromatin structure and inducing chromatin modifications, resulting in a change from heterochromatin to a permissive euchromatic state in this region. This transcription is both tissue- and time-specific and is responsible for the developmentally controlled expression of the *gh* gene, which promotes pituitary gland development [271]. SINE B1 elements also have insulator properties and can form heterochromatic barriers [272, 273]. It has been shown that B1 transcripts influence the chromatin state of proximal genes between embryonic stem cells and fibroblast cells, suggesting a primordial role of B1 elements in cell differentiation.

In addition to insulators, local chromatin structure is influenced by so called super-enhancers, which correspond to clusters of enhancers associated with Mediator complexes (transcriptional coactivators) that trigger the tissue-specific expression of genes [274]. A novel group of lncRNAs has recently been shown to interact with super-enhancers. These “super-lncRNAs” are able to form RNA:DNA:DNA triplex structures at specific sites within super-enhancers. Interestingly, approx. 40% of super-lncRNA binding sites in super-enhancers overlap with TEs, with SINEs and particularly Alu elements being the major contributors [274]. Moreover, it has been demonstrated that some lncRNAs can act as platforms interacting with several proteins and DNA [275]. For example, *Xist* lncRNAs can recruit Polycomb repression complex 2 [276] and also possess regions necessary for binding to DNA and transcriptional silencing [277, 278]. Thus, super-lncRNAs can possibly transport major regulators such as transcription factors and Mediator complexes to super-enhancers, influencing chromatin organization and driving surrounding tissue-specific gene expression.

## Conclusions

**Fig. 4:**
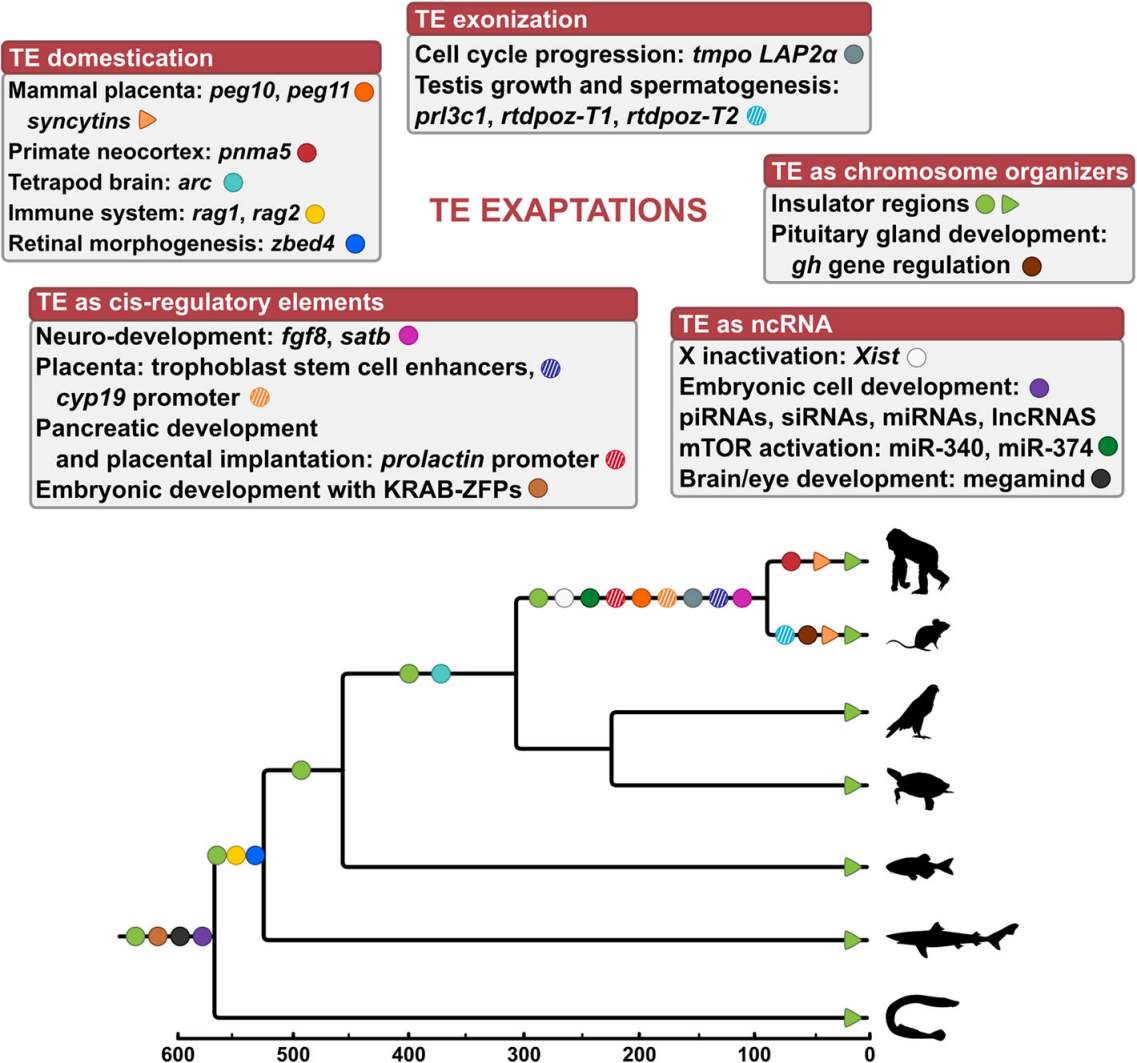
Timing of recruitment of selected TE-derived sequences in vertebrate development. Selected examples are summarized in boxes corresponding to the different types of co-option. These examples are plotted with colored dots onto the vertebrate phylogeny, indicating their timing of appearance and phylogenetic distribution (circles correspond to ancestral events with orthologous sequences in the species, triangles correspond to convergent events). Silhouette images from http://phylopic.org.

In this review, we present an overview of the multiple TE resources and functionalities that can be co-opted by host genomes (Fig. 4). TEs can be the source of developmental innovations through their recruitment as new coding sequences and new ncRNAs, and by acting as regulatory sequences, even if TEs are probably less active in gene regulation than expected from their abundance in vertebrate genomes [215]. Particularly, TEs have been instrumental to the evolution of brain, placenta, immunity and embryonic development in vertebrates. The pace of TE recruitment in vertebrate developmental program remains to be investigated. According to the developmental gene hypothesis for punctuated equilibrium, developmental regulatory genes essential for organism morphogenesis are extremely conserved and intolerant to mutations, maintaining an equilibrium state [279]. Changes might not be progressive but rather punctuated, this being often due to transposable elements accumulation and co-option as regulatory sequences to give rise to bursts of morphological innovations and species divergence.

Concerning the formation of new genes, Ohno proposed in 1999 that gene duplication is the main mechanism shaping evolutionary transitions [33]. New genes can also be formed from scratch, but this mechanism is very rare. We show here that TEs are a major source of material for the birth of novel protein-coding and RNA genes. In the absence of events of whole genome duplications, it has been estimated in primates that 53% of new genes originate at least partially from TE exaptation (mostly in primate-specific regions) compared to 24% from gene duplication and 5.5% de novo from non-coding sequences (the origin of the last 17.5% is still unclear) [280]. The contribution of TEs in this process is thus quantitatively important, in addition to the new functions they provide to the genome.

Several characteristics could modulate the propensity of TEs to be exapted. First, the different characteristics of each TE, such as the presence/absence of internal promoters, protein-binding motifs and ORFs encoding proteins with various properties, might favor the domestication of certain families depending on the needs of the host. For instance, ERVs have greater capacities to become gene regulatory drivers than most other TE families [215]. This has been proposed to be linked to the frequent loss of functional internal genes in ERVs, which abolish their transposition ability but leaves LTRs in genomes that can be readily repurposed. ERVs are frequently non-repressed in hypomethylated tissues, this also possibly facilitates their recruitment. Second, the age of the TE sequences might also be of importance. Repressive silencing being relaxed in old TEs, the repression of younger elements in the genome might limit their chance to be recruited by the host. Third, the activity, copy number and diversity of a TE family probably influence its evolutionary potential for the host. Even if low copy number elements can also lead to important innovations, as shown for the *Izanagi* transposon in the sex determination cascade of the medaka fish [236], high copy number and diversity of TEs might increase the probability of generating an element advantageous for the host at both sequence and localization levels. On the other hand, maintenance of transposition activity and recombination opportunity with other TE copies might hinder the fixation of a beneficial TE-derived sequence at a specific position in the genome. Fourth, the insertion preferences of TEs or the strength of the selection pressure against their maintenance certainly impact their possible recruitment. While TEs inserting or better tolerated in gene-poor regions will probably undergo less counter-selection, they might be often silenced in heterochromatin. On the other hand, TE preferential insertion or tolerance in gene-rich regions might be more frequently deleterious but could also increase the chance of generating a beneficial combination between TE and host sequences [27]. This might for example be the case for Alu elements in primates, which are probably better tolerated than LINEs in gene-rich regions due to their smaller size and therefore more frequently recruited in exaptation processes. The major factor influencing the co-option of a TE is probably the context of its insertion, as proposed for the domestication of the *Transib-*like DNA transposon at the origin of the V(D)J recombination [281]. A significant part (36.5% in the human genome) of TE-derived genes are positioned head-to-head to a host gene and share with him a bidirectional promoter containing a CpG island [282]. Since CpG islands correspond to open and actively transcribed chromatin regions, these promoters could be targeted by TE insertions and would provide them with a permissive transcriptional context for their expression, favoring the TE recruitment by the host as new transcribed sequences. TE domestication might also be facilitated by an insertion close to a promoter, or when the insertion results in a fusion with a host gene, with the TE possibly benefiting from the regulatory elements of the linked host gene if this gene is expressed in the germ line [64, 283, 284]. Fifth, if a novel TE is acquired by horizontal transfer, it will transiently escape the repression mechanisms of the host, bringing new evolutionary potentialities and recruitment opportunities.

Developmental pathways are closely linked to those causing cancer. Illustrating this, several examples of TE-derived developmental innovations have also been associated to cancer formation. The human *syncytin-1* gene, involved in immunomodulation and cell-cell fusion in placenta, is expressed in several cancers such as colorectal and breast cancers, and endometrial carcinoma [285–287]. Several genes of the PNMA family have also been implicated in cancers, such as *pnma5* or *pnma7a*, which acts as an oncogene in thyroid cancers [288, 289]. Finally, the RAG1/RAG2 recombinase, which catalyzes the V(D)J recombination, is a driver of the genetic instability linked to lymphoblastic leukemia [290].

To conclude, Barbara McClintock’s initial model [1] is now widely illustrated. In addition to form “controlling elements”, TEs are also a rich source of new host coding and RNA sequences. Most current examples illustrating the role of TE-derived sequences in vertebrate developmental innovation stems from mammals, but it is reasonable to think that TEs play also a major role in the evolution of other vertebrate species, which generally present even a higher diversity of transposable elements compared to mammals [21]. More studies in other vertebrate sub-lineages are therefore needed. For instance, an accumulation of TE sequences in the *Hox* gene clusters has been recently reported in four species of squamates (green-anole lizard, slow-worm, corn snake and gecko), which contrasts with the extremely conserved structure of *Hox* clusters in other vertebrates [291, 292]. It has been suggested that these TEs may provide new coding and non-coding regions or novel regulations of transcription to the cluster genes. The emergence of such elements inside the *Hox* clusters may explain the observed morphological diversity of squamates, but this hypothesis must now be tested at the functional level [292, 293]. The accurate characterization of the whole mobilome of multiple and divergent vertebrate species, i.e. the accurate and complete genome-wide identification and annotation of TEs and TE-derived sequences in genomes along with their evolutionary and functional characteristics, is an ongoing challenge that will allow to better assess the impact of TEs on vertebrate evolution.

## Data Availability

Not applicable.

## Abbreviations

2C: Two-Cell stage
ERV: Endogenous Retroviruse
HERV: Human Endogenous RetroVirus
JSRV: Jaagsiekte Sheep Retrovirus
KRAB-ZFP: Kruppel-associated box zinc finger proteins
lincRNA: long intergenic non-coding RNAs
LINE: Long Interspersed Nuclear Elements
lncRNA: long non-coding RNAs
LTR: Long Terminal Repeat
MER: Medium Reiteration frequency
MIR: Mammalian-wide Interspersed Repeat
miRNA: microRNA
MITE: Miniature Inverted Repeat Transposable Element
nt: nucleotide
ORF: Open Reading Frame
piRNA: PIWI-interacting RNAs
RSS: Recombination Signal Sequence
RT: Reverse Transcriptase
SINE: Short Interspersed Nuclear Elements
siteRNA: small intronic transposable element RNA
sncRNA: small non-coding RNA
TAD: Topologically Associated Domains
TBP: TATA box-binding protein
TE: Transposable Element
TF: Transcription Factor
TFBS: Transcription Factor Binding Site
TIR: Terminal Inverted Repeat
TSC: Trophoblast Stem Cell
UTR: Untranslated Region
